# Excellent Bitters

**Published:** 1886-10

**Authors:** 


### *EXCELLENT BITTERS.*

We are not aware that the following recipe for bitters has ever before been published, but we are able to recommend it as one of the very best:

One pint alcohol, 1 ounce gentian, powdered; 1 ounce cardamon seeds, 1 ounce orange peel, powdered. Shake up and let stand three days, then draw off for use.

**The consumption of Tea.**—Great Britain alone consumes eighty million pounds of it annually, and the United States about seventy million pounds in the same time.

